# Supplementary material for: Auditory processing remains sensitive to environmental experience during adolescence in a rodent model
Source: Nat Commun. 2022 May 24;13:2872. doi: 10.1038/s41467-022-30455-9 (PMC9130260; doi:10.1038/s41467-022-30455-9)
Supplement: Supplementary file 1 — Supplementary Figures [file 41467_2022_30455_MOESM1_ESM.pdf]

Supplementary Figures

Auditory processing remains sensitive to environmental experience during adolescence in a rodent model

Anbuhl KL, Yao JD, Hotz RA, Mowery TM, Sanes DH

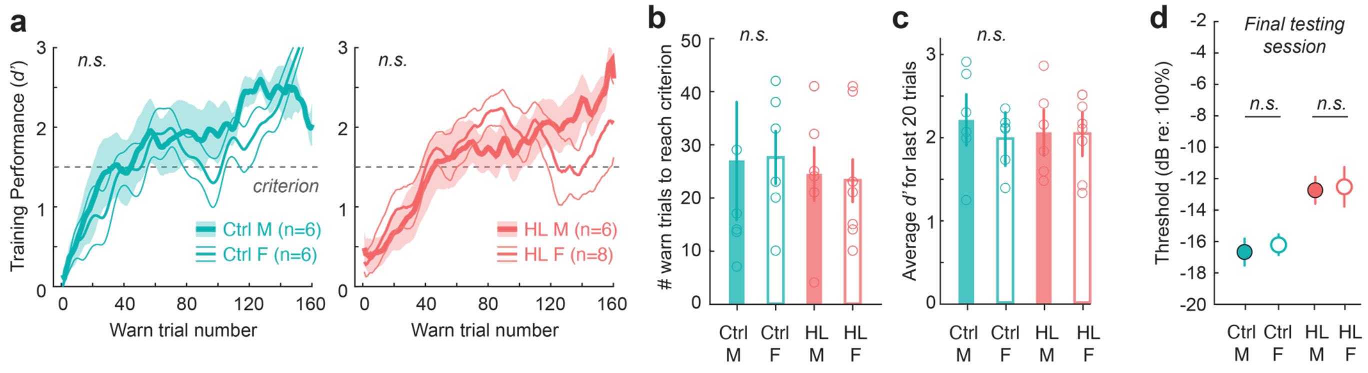

Supplemental Figure 1. Transient hearing loss during adolescence did not alter procedural training or psychometric testing performance in a sex-specific manner. (**a**) Procedural training sessions (3-4 separate sessions) were combined separately for male (M) and female (F) control (Ctrl; left panel) and adolescent hearing loss (HL; right panel) animals. Behavioral performance (*d'*) was computed as a function of warn trial number using a 5-trial sliding window. Data are depicted as the mean ± SEM. There are no significant differences between males and females for control or HL groups ($p = 0.61$, two-way mixed model ANOVA). (**b**) Number of warn trials to reach performance criterion ($d' \geq 1.5$) are not significantly different for male (M) and female (F) control (Ctrl) and adolescent HL animals ($p = 0.97$, two-way mixed model ANOVA). Circles indicate individual values, and the error bars indicate mean ± SEM. (**c**) Average *d'* for the last 20 trials of procedural training for male and female control and HL animals are not significantly different from one another ($p = 0.55$, two-way mixed model ANOVA). Circles indicate individual values, and the error bars indicate mean ± SEM. (**d**) Control (Ctrl) and adolescent hearing loss (HL) males (M; closed circles) and females (F; open circles) exhibit comparable AM detection thresholds on the final testing session, with no significant differences between males or females for controls ($p = 0.81$) or HL animals ($p = 0.78$; two-way mixed model ANOVA followed by Tukey's test for multiple comparisons). Data are presented as mean ± SEM. Therefore, transient hearing loss during adolescence impairs amplitude modulation (AM) depth detection for both male and female adults equally.

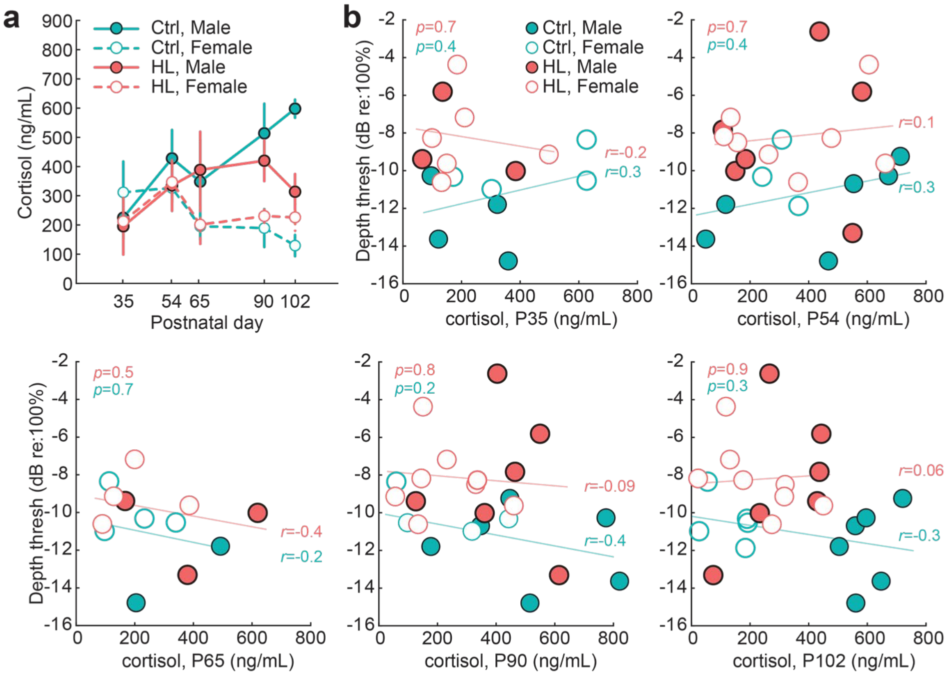

Supplementary Figure 2. Poorer behavioral detection thresholds are not due to elevated stress during adolescence. (**a**) Serum cortisol levels across age for male (solid line) and female (dotted line) gerbils. For each animal, there were 3-5 cortisol assessments at timepoints spanning P35-P102 (Control M: n = 6 subjects, 25 total samples; Control F: n = 6 subjects, 25 total samples; HL M: n = 6 subjects, 24 total samples; HL F: n = 8, 34 total samples). Circles and error bars indicate the mean ± SEM for each condition. (**b**) Behavioral depth thresholds for the first day of perceptual testing (~P126; dB re: 100%) as a function of cortisol levels collected at P35, P54, P65, P90, and P102 for individual subjects. Solid line indicates the linear fit for each group (Control, adolescent HL), along with the associated two-tailed Pearson's *r* correlation value. The *p*-values of each linear fit are listed in the top left corner of each plot. Elevated cortisol levels at any of the ages collected do not correlate with poorer detection thresholds at P126.

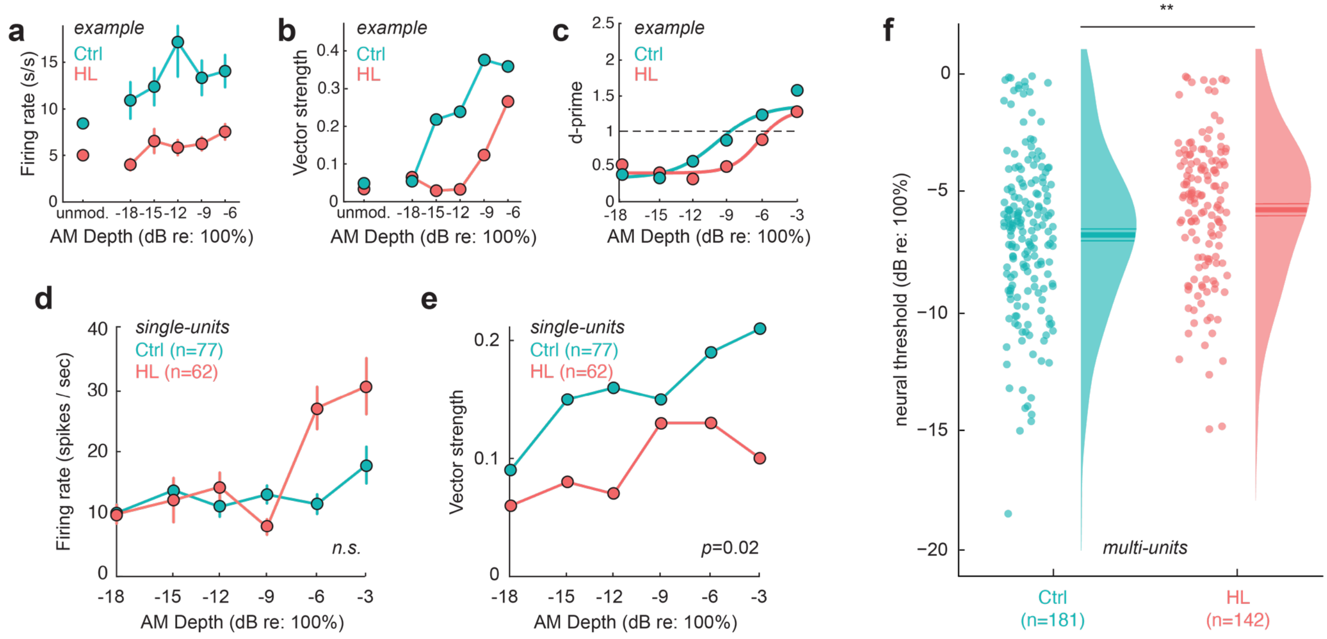

Supplementary Figure 3. Basic response properties and detection thresholds for individual auditory cortex neurons. (**a-c**) Firing rate (s/s), vector strength (VS), and *d'* values for two example single units from the Control and HL examples shown in Figure 5c. The circles and associated error bars in **a** depict the mean ± SEM firing rate (s/s) for all trials for each depth within one behavior session. (**d-e**) Firing rate (s/s) and vector strength for a population of single units that met the criteria for AM sensitivity (same neurons from Figure 5d-f). The circles and associated error bars in **d** depict the mean ± SEM firing rate (s/s) for all single units at each depth across all behavior sessions. There is no effect of adolescent HL on the firing rate of single units (**d**; $p = 0.07$, two-way mixed model ANOVA), but there is an effect on vector strength (**e**; $p = 0.02$, two-way mixed model ANOVA). (**f**) Neural thresholds for multi-units are plotted for control (n = 181 units) and HL animals (n = 142 units). Individual thresholds are shown (circles), along with a half-violin plot indicating the probability density function. Horizontal lines indicate the mean ± SEM. Individual multi units from HL animals exhibit poorer neural depth thresholds than control single units ($p = 0.003$, two-way mixed model ANOVA).

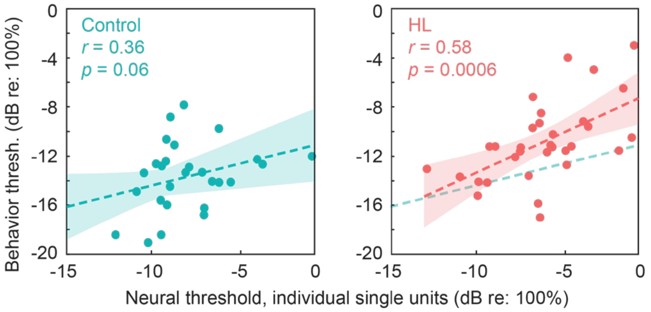

Supplementary Figure 4. Neural sensitivity of individual cortical neurons correlates with perceptual performance. Behavioral threshold as a function of neural thresholds for individual single units that meet the criteria for AM sensitivity (see Methods). The neural thresholds are the average threshold for single units per session (i.e., 1 avg / session). There are a total of 28 awake-behaving sessions for Controls, and 32 awake-behaving sessions for HL animals. Dotted lines indicate a fitted linear regression, with shaded areas indicating the (± 1 SD) of the prediction error. The two-tailed Pearson's r and statistical significance of each fit are noted in the top left corner of each plot. There is a positive correlation between behavioral and single-unit neural thresholds for control ($r = 0.36$, $p = 0.06$) and HL animals ($r = 0.58$, $p = 0.0006$).
