# Supplementary material for: Auditory processing remains sensitive to environmental experience during adolescence in a rodent model
Source: Nat Commun. 2022 May 24;13:2872. doi: 10.1038/s41467-022-30455-9 (PMC9130260; doi:10.1038/s41467-022-30455-9)
Supplement: Supplementary file 2 — Reporting Summary [file 41467_2022_30455_MOESM2_ESM.pdf]

nature portfolio

Corresponding author(s): KL Anbuhl, DH Sanes

Last updated by author(s): 04/19/2022

Reporting Summary

Nature Portfolio wishes to improve the reproducibility of the work that we publish. This form provides structure for consistency and transparency in reporting. For further information on Nature Portfolio policies, see our Editorial Policies and the Editorial Policy Checklist.

Statistics

For all statistical analyses, confirm that the following items are present in the figure legend, table legend, main text, or Methods section.

n/a	Confirmed	
☐	☒	The exact sample size (*n*) for each experimental group/condition, given as a discrete number and unit of measurement
☐	☒	A statement on whether measurements were taken from distinct samples or whether the same sample was measured repeatedly
☐	☒	The statistical test(s) used AND whether they are one- or two-sided<br>*Only common tests should be described solely by name; describe more complex techniques in the Methods section.*
☐	☒	A description of all covariates tested
☐	☒	A description of any assumptions or corrections, such as tests of normality and adjustment for multiple comparisons
☐	☒	A full description of the statistical parameters including central tendency (e.g. means) or other basic estimates (e.g. regression coefficient) AND variation (e.g. standard deviation) or associated estimates of uncertainty (e.g. confidence intervals)
☐	☒	For null hypothesis testing, the test statistic (e.g. *F*, *t*, *r*) with confidence intervals, effect sizes, degrees of freedom and *P* value noted<br>*Give P values as exact values whenever suitable.*
☒	☐	For Bayesian analysis, information on the choice of priors and Markov chain Monte Carlo settings
☒	☐	For hierarchical and complex designs, identification of the appropriate level for tests and full reporting of outcomes
☐	☒	Estimates of effect sizes (e.g. Cohen's *d*, Pearson's *r*), indicating how they were calculated

Our web collection on statistics for biologists contains articles on many of the points above.

Software and code

Policy information about availability of computer code

Data collection	More detail regarding hardware and software used for data collection can be found in the Methods section.<br><br>All behavioral data were collected using custom MATLAB scripts developed by Dr. Daniel Stolzberg (https://github.com/dstolz/epsych) using Tucker-Davis Technologies hardware. In vivo electrophysiological recordings were collected using a wireless recording system (Triangle BioSystems) and the Tucker-Davis Technologies Synapse Suite (https://www.tdt.com/component/synapse-software/). All in vitro physiology data were acquired using a custom-designed IGOR (version 4.08; WaveMetrics, Lake Oswego, OR) macro (Slice™; http://www.cns.nyu.edu/~sanes/slice_software/). Hormones were analyzed on a Shimadzu Nexera-LCMS-8050 LC-MS/MS platform at the Endocrine Technologies Core, Oregon National Primate Research Center (ONRPC).
Data analysis	All data were analyzed using open access software and custom MATLAB or Python scripts. Statistical analyses and procedures were performed using JMP Pro 16.0 (SAS) or custom-written MATLAB (MathWorks, R2019b) scripts utilizing the Statistics and Curve Fitting Toolboxes. Psychometric fitting was performed using Psignifit4 (https://github.com/wichmann-lab/psignifit). Semi-automatic spike sorting was performed using Kilosort (https://github.com/cortex-lab/KiloSort) and manually inspected and refined in Phy (https://github.com/cortex-lab/phy). In vitro physiology brainslice data were analyzed offline using a second Igor Macro (Slice Analysis: http://www.cns.nyu.edu/~sanes/slice_software/). Auditory brainstem responses (ABRs) were analyzed using custom python scripts provided by Brandon Warren and Edwin Rubel (University of Washington). Custom scripts used for data analysis can either be found in the Methods section, a public repository (https://nyu.box.com/v/anbuhl-et-al-2022) or will be made available upon email request to the corresponding author, Kelsey Anbuhl: kla7@nyu.edu).

For manuscripts utilizing custom algorithms or software that are central to the research but not yet described in published literature, software must be made available to editors and reviewers. We strongly encourage code deposition in a community repository (e.g. GitHub). See the Nature Portfolio guidelines for submitting code & software for further information.

Data

Policy information about availability of data

All manuscripts must include a data availability statement. This statement should provide the following information, where applicable:

- Accession codes, unique identifiers, or web links for publicly available datasets
- A description of any restrictions on data availability
- For clinical datasets or third party data, please ensure that the statement adheres to our policy

Source data are available with this paper. Additional raw data are available on a public repository (https://nyu.box.com/v/anbuhl-et-al-2022).

Field-specific reporting

Please select the one below that is the best fit for your research. If you are not sure, read the appropriate sections before making your selection.

☒ Life sciences ☐ Behavioural & social sciences ☐ Ecological, evolutionary & environmental sciences

For a reference copy of the document with all sections, see nature.com/documents/nr-reporting-summary-flat.pdf

Life sciences study design

All studies must disclose on these points even when the disclosure is negative.

Sample size

Sample size was determined based on previous behavioral, in vivo and in vitro physiology studies published from our laboratory and associated power analyses that provided a minimum number of subjects required per group to establish significance (8-10 subjects). With the exception of the control behavioral data collected for Figure 9, all behaviorally-trained animals for the adolescent hearing loss experiment underwent ABR testing and were used for in vivo or in vitro physiology experiments. For the adolescent hearing loss experiment: Unless stated otherwise, all control groups contained 12 normal-hearing animals and all experimental groups contained 14 animals that experienced adolescent hearing loss. Both groups came from 4 separate litters; control and adolescent hearing loss animals were divided evenly within each litter. For the Adult-onset hearing loss experiment: All Adult controls contained 8 normal-hearing animals and all experimental groups contained 8 animals that experienced adult-onset hearing loss. Both groups came from 4 separate litters; control and adult-onset hearing loss animals were divided evenly within each litter.

Data exclusions

No animals were removed from behavioral or in vitro physiology experiments. In vivo physiology data was not included if the electrodes were not targeting core auditory cortex, or if neural responses did not meet specific auditory response criteria (specified in the Methods). All animals received ABR measurements, however ABR data collection for one adolescent hearing loss animal ended prematurely due to failure to keep animal sufficiently anesthetized during assessment. For hormone data collection, some blood collection time points yielded too little serum volume to adequately perform hormone quantification. Finally, though serum samples were tested for estradiol levels at all ages (P35, P54, P65, P90, P102), the analysis equipment used failed to detect estradiol levels at ages P35, P54, and P90 due to lack of sensitivity.

All exclusion criteria were established prior to data collection.

Replication

All experiments were replicated in multiple animals (Adolescent hearing loss experiment: 12 control animals and 14 experimental animals from 4 different litters; Adult-onset hearing loss experiment: 8 control animals and 8 experimental animals from 4 different litters). Unless explicitly stated, all numbers of subjects are listed in the figures or the figure legends.

Randomization

All subjects within a litter were randomly assigned to one of two groups (Adolescent hearing loss experiment: control or adolescent hearing loss group; Adult-onset hearing loss experiment: adult control or adult-onset hearing loss group) with the following exception: care was taken to ensure equal distribution of male and female pups within each group assignment.

Blinding

Investigators were blind to the group assignment for hormone and in vitro physiology data collection and analysis. For behavior, in vivo physiology and ABR collection and analysis, blinding was not relevant as identical criteria, analysis pipeline and unbiased statistical tests were used for all data.

Reporting for specific materials, systems and methods

We require information from authors about some types of materials, experimental systems and methods used in many studies. Here, indicate whether each material, system or method listed is relevant to your study. If you are not sure if a list item applies to your research, read the appropriate section before selecting a response.

Materials & experimental systems

n/a	Involved in the study
☒	☐ Antibodies
☒	☐ Eukaryotic cell lines
☒	☐ Palaeontology and archaeology
☐	☒ Animals and other organisms
☒	☐ Human research participants
☒	☐ Clinical data
☐	☐ Dual use research of concern

Methods

n/a	Involved in the study
☒	☐ ChIP-seq
☒	☐ Flow cytometry
☒	☐ MRI-based neuroimaging

Animals and other organisms

Policy information about studies involving animals; ARRIVE guidelines recommended for reporting animal research

Laboratory animals	Mongolian gerbils of equal distribution of each sex were used in the study. Manipulations were performed in developing gerbils from postnatal (P) day 23 through P102. All hormone collections were performed between P35 and 102. All behavioral, ABR, in vitro and in vivo physiological measures were obtained in adult gerbils (>P123). For the adult-onset hearing loss experiment, manipulations were performed in adult gerbils (>P102) for 80 days. All behavioral measures were obtained in adult gerbils (>P203).
Wild animals	No wild animals were used in this study.
Field-collected samples	The study did not involve samples collected in the field.
Ethics oversight	All procedures were approved by the Institutional Animal Care and Use Committee at New York University.

Note that full information on the approval of the study protocol must also be provided in the manuscript.

Dual use research of concern

Policy information about dual use research of concern

Hazards

Could the accidental, deliberate or reckless misuse of agents or technologies generated in the work, or the application of information presented in the manuscript, pose a threat to:

No	Yes
☒	☐ Public health
☒	☐ National security
☒	☐ Crops and/or livestock
☒	☐ Ecosystems
☒	☐ Any other significant area

Experiments of concern

Does the work involve any of these experiments of concern:

No	Yes
☒	☐ Demonstrate how to render a vaccine ineffective
☒	☐ Confer resistance to therapeutically useful antibiotics or antiviral agents
☒	☐ Enhance the virulence of a pathogen or render a nonpathogen virulent
☒	☐ Increase transmissibility of a pathogen
☒	☐ Alter the host range of a pathogen
☒	☐ Enable evasion of diagnostic/detection modalities
☒	☐ Enable the weaponization of a biological agent or toxin
☒	☐ Any other potentially harmful combination of experiments and agents
